# Pterocellin A isolated from marine bryozoan *Pterocella vesiculosa* is cytotoxic to human HeLa cells via mitochondrial apoptotic processes

**DOI:** 10.1186/s40064-016-2397-9

**Published:** 2016-06-16

**Authors:** Alice T. Wang, Michèle R. Prinsep, Ryan D. Martinus

**Affiliations:** School of Science, Faculty of Science and Engineering, The University of Waikato, Hamilton, New Zealand

## Abstract

Pterocellin A is a novel bioactive alkaloid isolated from the New Zealand marine bryozoan *Pterocella vesiculosa*. It exhibits potent antitumour activity towards the P388 (murine leukaemia) cell line in vitro and is selectively sensitive towards certain non-small cell lung, melanoma, and breast cancer cell lines, however, the biological mode of action of pterocellin A is unknown. Using the human cervical cancer cell line HeLa, we show that pterocellin A exhibited cytotoxicity against HeLa cells with an IC_50_ of 886 ng/mL. Time-course MTT and LDH assays were carried out and the results showed only a low level of cytosolic LDH was detected in the supernatant after all the cells have died from pterocellin A treatment at 2000 ng/mL. This indicated the cells maintained membrane integrity upon cell death which suggested apoptotic cell death. Additionally, morphological changes were observed under the microscope after 6 h of treatment. Cell shrinkage and nucleus condensation were observed, as well as apparent membrane blebbing, a key feature of apoptosis. The MTT data was also indicative of mitochondria impairment which could suggest that pterocellin A targets the mitochondria. This idea was supported by the observed changes in the morphology and location of the mitochondria after exposure to pterocellin A. Furthermore, the level of activated caspase-3 in HeLa cells increased after treatment with pterocellin A; activated caspase-3 can only be detected after a series of signalling events following the induction of apoptosis. These data support the notion that pterocellin A is an inducer of apoptosis in HeLa cells possibly via mitochondria related processes.

## Background

Natural products, also known as secondary metabolites, are organic compounds produced by living organisms. Unlike primary metabolites such as carbohydrates and amino acids, secondary metabolites are considered non-essential to life and have no apparent function within the organism (Williams et al. 1989). These compounds attract research interest because they can exert physiological effects on other organisms, often playing an ecological role in regulating a wide range of chemical interactions. These include pheromone chemical communication, chemical defence mechanisms, and mutualistic interactions between plants, microorganisms, insects and animals (Verpoorte 1998). Natural products are known for their characteristic chemical diversity, novelty and structural complexity, often containing unusual combinations of functional groups which attribute to biochemical specificity and other molecular properties (Koehn and Carter 2005). Many bioactive compounds have been found to have therapeutic effects in humans. These properties thus make natural products ideal candidates for lead compounds in drug discovery in the pharmaceutical industry.

The rate of discovery of novel bioactive compounds from terrestrial organisms has decreased in recent years, due to an increased frequency of rediscovery (Dias et al. 2012). This led to the search being extended to unexplored habitats in marine environments. The marine environment is a rich source of biological and chemical biodiversity. The ocean covers greater than 70 % of the earth’s surface but the chemical resources from this environment are largely untapped (Donia and Hamann 2003). Thirty-two of the thirty-three animal phyla are represented in aquatic environments and fifteen phyla are exclusive to the marine environment (Colegate 2007). Potential pharmaceutical leads from natural products are likely to be found in places with high biodiversity, such as the ocean fringe or coral reefs in marine environments (Hay and Fenical 1996). Marine natural products are often produced in sessile, colonial marine species such as soft-bodied invertebrates including sponges, bryozoans, and tunicates. These organisms, living in such a highly competitive environment, have evolved to overcome the ecological pressure to compete for space with surrounding species and to evade predators (Van Minh et al. 2005). Due to their lack of movement and physical defence structures, it is thought that these organisms survive by utilising natural products as chemical defence mechanisms (Beesoo et al. 2014).

Bryozoans, commonly known as sea moss or moss animals, are sedentary, filter-feeding invertebrates found in aquatic environments. There are over 8000 species of bryozoans described, many more were observed in fossil records (Pechenik 2014). Most species inhabit marine environments with very few in freshwater lakes and rivers (Ryland 1970). Bryozoans are of particular research interest due to the variety of secondary metabolites they contain. A number of novel natural products from bryozoans have been isolated to date. These have been extensively reviewed (Blunt et al. 2015; Sharp et al. 2007).

**Fig. 1:**
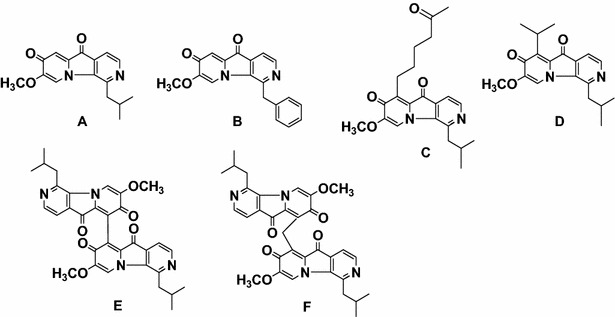
Structures of Pterocellins A–F

Pterocellins are a class of novel alkaloids isolated from the marine bryozoan *Pterocella vesiculosa* collected off the coast of the North Island, New Zealand. Structures of pterocellins A–F (Fig. 1) have been elucidated by NMR spectroscopy and single-crystal X-ray diffraction (Prinsep 2008; Yao et al. 2003). Bioactivity testing of two pterocellins (A, B) indicated promising anti-bacterial, anti-tumour, and anti-fungal activities. It was found that pterocellins A and B were active against the P388 murine leukaemia cell line (IC50 values 477 and 323 ng/mL, respectively); non-small cell lung (NCI-H23), melanoma (MALME-3M, M14, SK-MEL-5), and breast (MDA-MB-435, MDA-N) cell lines (Yao et al. 2003). However, the mode of cytotoxicity of pterocellin A is not yet known. Since cytotoxic natural products that selectively target the apoptotic pathways of cancer cells are particularly of interest in the development of chemotherapeutic agents. We investigated to see if pterocellin A was cytotoxic to the human cervical cancer cell line HeLa via apoptotic cell death processes.

## Methods

### HeLa cell culture

The human cervical cancer cell line, HeLa, was purchased from the American Tissue Culture Collection (ATCC) (Number CCL-2). The cells were grown in DMEM (25 mM d-Glucose, 4 mM l-Glutamine, 1 mM sodium pyruvate, Gibco) supplemented with 100X units of penicillin/streptomycin (Gibco) and 10 % foetal bovine serum (FBS) (Gibco). Cells were grown in T-25 flasks (Biofil) in standard incubation conditions in a humidified incubator (Heraeus HERAcell™) at 37 °C with 5 % CO_2_. Cells were harvested and subcultured every 4 days at near confluency.

### MTT assay

The Sigma thiazolyl blue tetrazolium bromide (MTT) reagent was used as per the manufacturer’s protocol. The MTT solution was freshly prepared before each experiment by dissolving in complete DMEM at a concentration of 5 mg/mL, pre-warmed to 37 °C and passed through a filter (0.2 μm) with a syringe. The solubilising solution was also freshly prepared to a final concentration of 0.1 M HCl in isopropanol.

Each experiment was carried out in triplicate samples in 96-well cell culture plates (Biofil). There were three controls in each experiment: growth control, negative control and solvent control. Growth control consisted of untreated cells in media; solvent control consisted of untreated cells in media, with the addition of MeOH equal to the solvent percentage of the highest treatment concentration and the negative control consisted of media plus solvent. The final solvent concentration did not exceed 2 % of the total volume. Near confluent cells in log-phase were harvested and seeded at a density of 1 × 10^4^ cells per well, each well containing 150 μL cell culture. The cells were then incubated at standard conditions overnight to allow the cells to attach and recover from handling before treatment was added. To carry out the assay, existing media containing test compound were removed and replaced with fresh media (150 μL per well). This was followed by the addition of reconstituted MTT in an amount equal to 10 % of the media volume (15 μL) and incubation at standard conditions for 2 h. The MTT media was then carefully removed and replaced with MTT solubilising solution in an amount equal to the original volume (150 μL) to dissolve the formazan crystals. The samples were then placed on a plate-shaker (IKA® MS 1) for 15 min at room temperature, protected from light. Once homogenised, the samples were spectrophotometrically measured on a plate reader (Bio-Rad 680) at an absorbance wavelength of 570 nm, with a background absorbance reading at 655 nm.

### LDH assay

The LDH assays were carried out simultaneously to MTT assays using a CytoTox 96® Non-Radioactive Cytotoxicity Assay commercial kit (Promega) as per the manufacturer’s protocol. The assay involved taking supernatant from treated cells without disturbing the cell population for MTT assay. The substrate mix used in the assay was reconstituted with assay buffer (12 mL) and stored at −20 °C for no more than 6–8 weeks protected from light. There were several controls included in these experiments: spontaneous release control (SRC), maximum release control (MRC), and culture medium background control (CM). Spontaneous control corrected for the spontaneous release of LDH from cells; maximum control represented 100 % release of LDH upon cell lysis and culture medium background control corrected for any LDH activity contributed by FBS in the DMEM, and the varying amounts of phenol red in the media. The growth control from MTT assays served the same purpose as the spontaneous control in LDH assays.

Cells were incubated with test compound as per experimental protocol. Prior to harvesting supernatant, 15 μL of lysis solution (10X) was added to the maximum release control. This allowed total cell lysis and therefore the release of total LDH into the supernatant. After incubating for 45 min, the maximum release control was checked under the microscope to confirm complete lysis. The culture plate was then centrifuged (Heraeus™ Multifuge™ X3) at 250×*g* at room temperature for 4 min to pellet any cell debris.

To carry out the assay, 50 μL supernatant from each sample of the culture plate was transferred to a new assay plate (Greiner Bio-One). The reconstituted Substrate Mix was then brought to room temperature and 50 μL was added to each sample. This was incubated for 30 min at room temperature (protected from light using foil), followed by the addition of Stop Solution (50 μL) to each sample. The samples were measured on a plate reader (Bio-Rad 680) at an absorbance of 490 nm within an hour of adding the stop solution.

### Caspase-3(active) assay

This assay was carried out using a commercial kit Caspase-3 (active) FITC Staining Kit (Abcam) as per the manufacturer’s protocol with small modifications. Near confluent cells in log-phase were harvested and seeded at a density of 3 × 10^5^ in a 6-well cell culture plate (Greiner Bio-One). The cells were then incubated at standard incubation conditions overnight to allow the cells to attach and recover from handling before treatment was added. After treatment, the media from each well was transferred to Falcon tubes (15 mL) to collect any detached cells from treatment. The remaining cells were harvested by washing with pre-warmed PBS (1 mL) followed by the addition of trypsin/EDTA (0.5 mL). Once detached, pre-warmed DMEM (1.5 mL) was added to each well to neutralise trypsin before being transferred to the corresponding Falcon tubes. An aliquot (100 μL) was then taken from each sample for a cell count. The remaining cells were centrifuged at 800×*g* for 5 min, and the cell pellets were re-suspended in pre-warmed DMEM (1 mL). To measure the fluorescent activity of caspase-3, FITC-DEVD-FMK (1 μL) was added to aliquots (30 μL) of each sample in Eppendorf tubes (1.5 mL) and incubated at standard incubation conditions for 1 h. After incubation, the cells were centrifuged at 3000 rpm for 5 min and the supernatant was removed. Wash Buffer (0.5 mL) was then added followed by centrifugation; and this step was repeated. Finally, the cell pellet was re-suspended in Wash Buffer (100 μL) then transferred to a black microtitre plate (Greiner Bio-One). The fluorescence intensity was measured on a plate reader (BMG Fluostar Optima) at excitation and emission wavelengths of 485 and 520 nm respectively.

### Confocal fluorescent microscopy

Fluorescent microscopy work was carried out on an Olympus FV1000 laser scanning confocal microscope. The staining and visualisation of the mitochondria utilised a fluorescent dye, MitoTracker Red CMXRos which has peak absorption and emission at 578 and 599 nm respectively, producing a red fluorescence.

A stock solution (1 mM) was made up according to the manufacturer’s manual by adding DMSO (94 μL) to a vial (50 μg) of MitoTracker Red CMXRos at room temperature. An aliquot (10 μL) was made up to 1 μM with complete DMEM (9.99 mL) and stored in the dark at −20 °C. The working solutions (50 nM) were prepared fresh from the 1 μM stock solution before each experiment.

To visualise mitochondria under fluorescence, cells were grown in a Fluorodish™ (WPI) and stained with working solution under standard conditions for 20 min. The staining media was then replaced with fresh media and cells were incubated for a further 10 min to wash away unincorporated dye.

### Statistical analysis

All statistical analyses were carried out using Microsoft Excel. Data was averaged where appropriate, and the standard error of the mean (S.E.M) was calculated using Excel. A two-tailed Student’s t test was carried out to determine the significance of the data, and the accepted level of significance was p < 0.05, which was denoted as “*”.

## Results

**Fig. 2:**
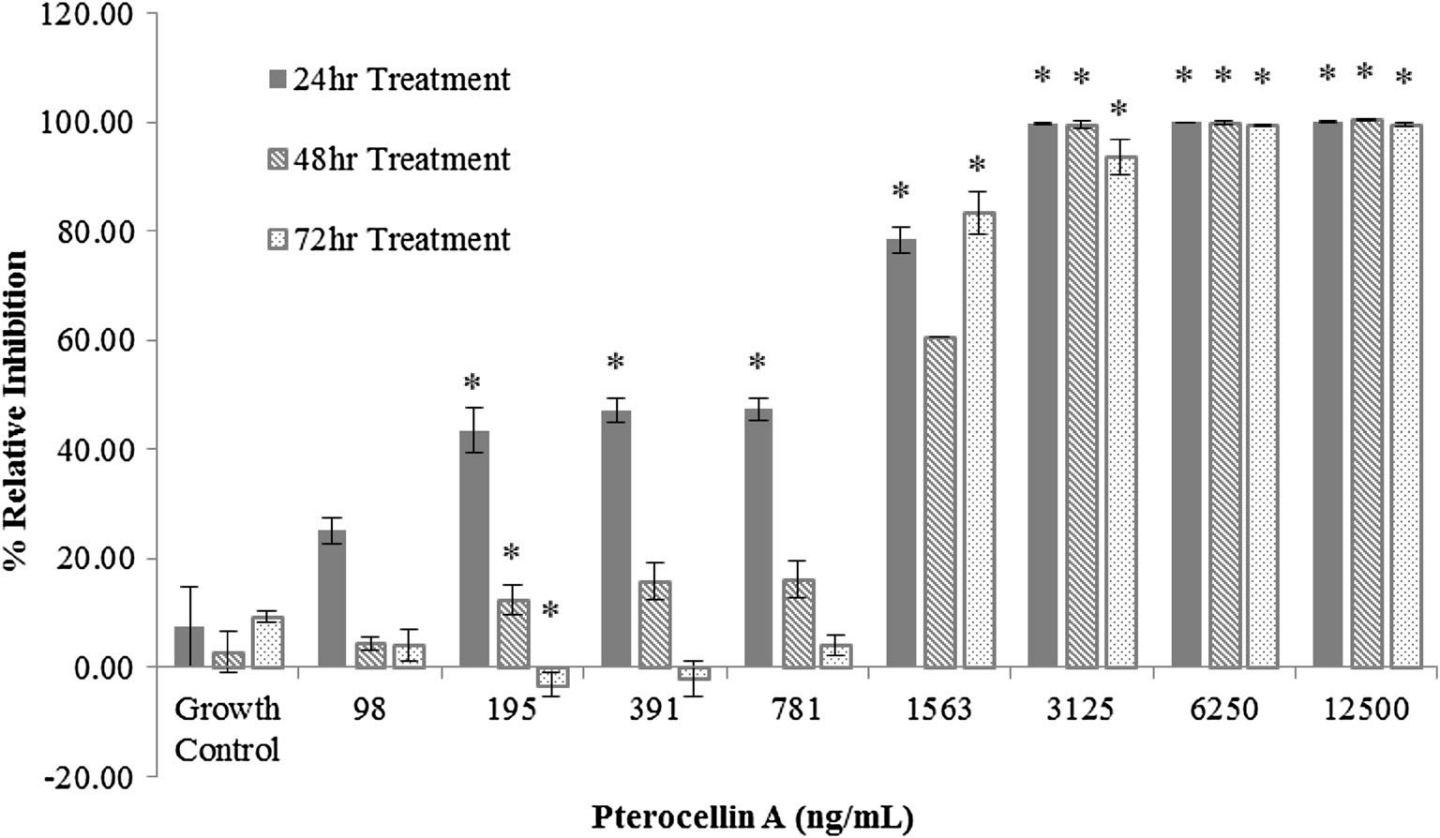
Pterocellin A toxicity against HeLa cells after 24, 48 and 72 h treatment, MTT assay. *Bar graph* showing the mean ± S.E.M, *p < 0.05, N = 3

After 24 h incubation, HeLa cells treated with pterocellin A had an IC_50_ value of 886 ng/mL, which was within the same order of magnitude as the IC_50_ of pterocellin A with the P388 cell line noted in a previous study (Yao et al. 2003). Interestingly the relative inhibition of cells treated with the lower concentrations decreased over this time which attributed to the higher IC_50_ values (Fig. 2). This suggests the cells were able to recover and overcome the cytotoxic effects of pterocellin A after a period of time.

**Fig. 3:**
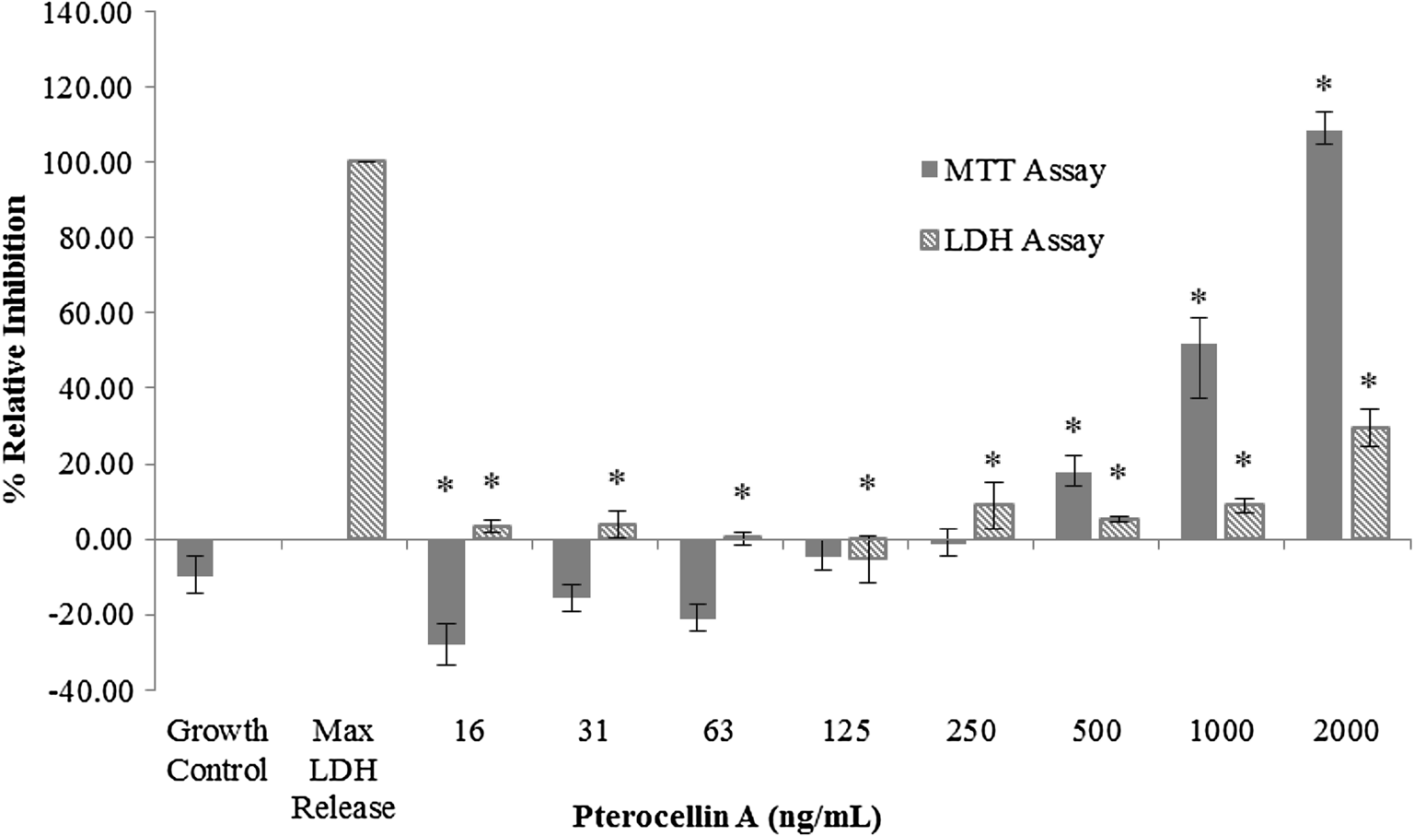
Pterocellin A toxicity against HeLa cells at 24 h treatment, MTT and LDH assays. *Bar graph* showing the mean ± S.E.M, *p < 0.05, N (MTT) = 12; N (LDH) = 6

LDH assays were carried out to find out if the decrease in cell viability affected the plasma membrane integrity. Based on the MTT assay, it was found that there was a steady increase in relative inhibition as the concentration increased, however the level of detected LDH remained relatively low. All cells were killed at 2000 ng/mL after 24 h, and the amount of LDH released was 29.54 % (Fig. 3).

**Fig. 4:**
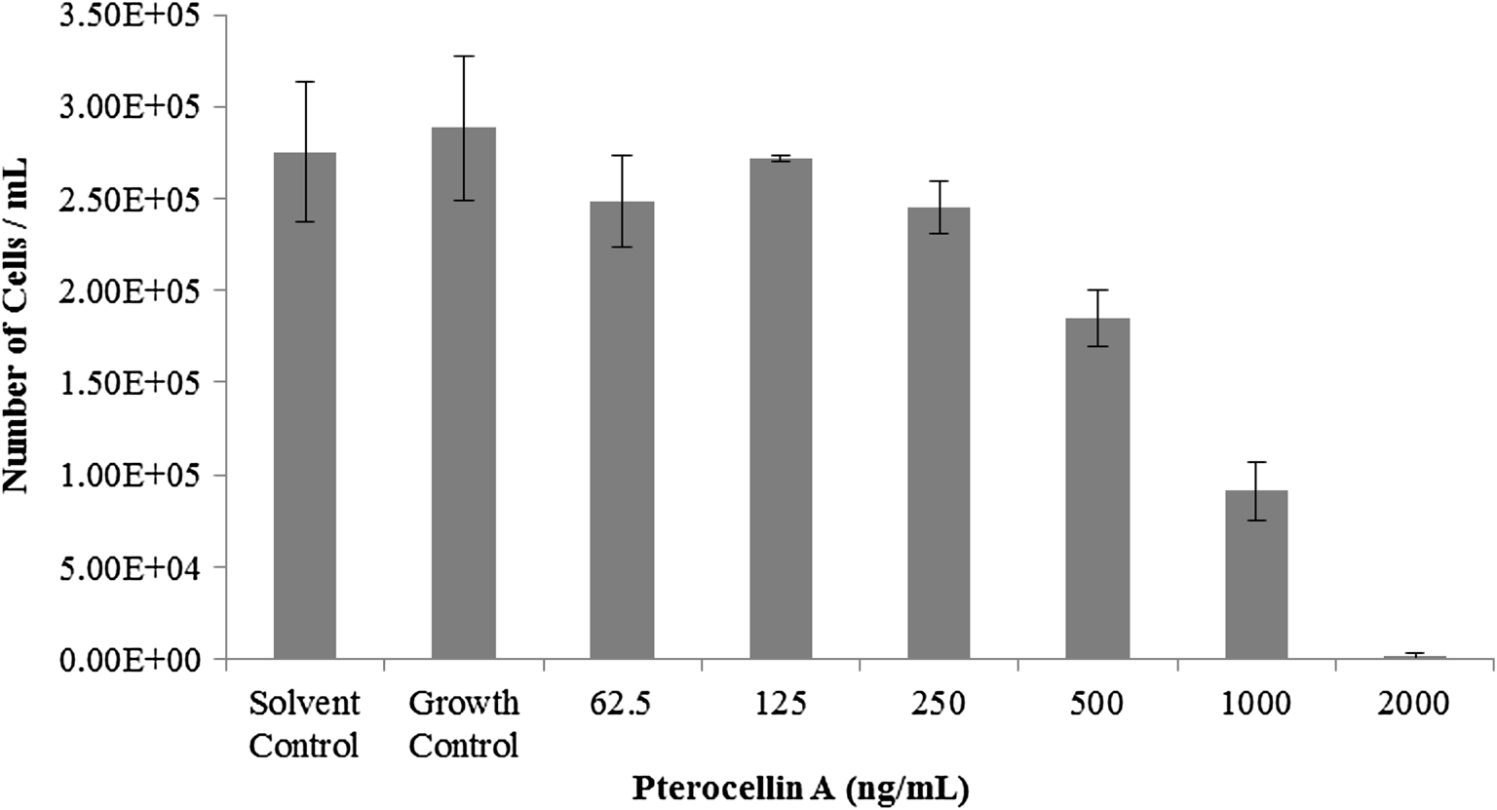
Trypan blue exclusion cell count after 24 h treatment. *Bar graph* showing the mean ± S.E.M, N = 3

A basic trypan blue exclusion cell count can distinguish between dead and live cells. Stained cells observed under the microscope would indicate a cell with a compromised plasma membrane. A trypan blue exclusion cell count was carried out after treating cells with different concentrations of pterocellin A for 24 h. It was found that no stained cells were observed after treatment, the cell number steadily decreased as the concentration of pterocellin A increased (Fig. 4).

**Fig. 5:**
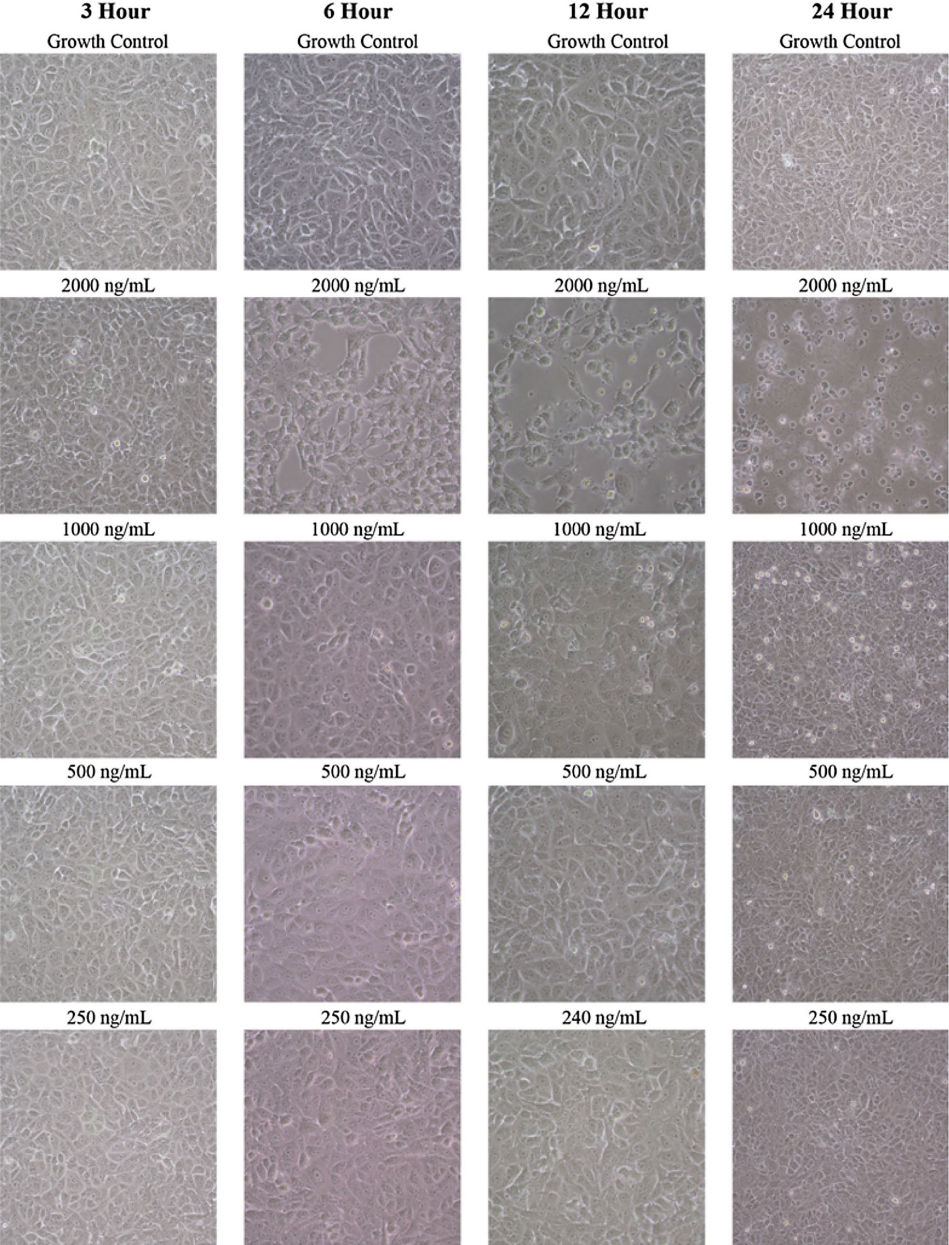
Images of cells treated at different concentrations of pterocellin A at different times Photos taken at ×200 magnification using an inverted Nikon eclipse TS100 microscope with a Nikon COOLPIX 4500 camera

The morphology of the cells started to change after 6 h of incubation in the presence of pterocellin A (Fig. 5). Compared to the untreated normal cells which appeared elongated and had contact with neighbouring cells, cells treated with pterocellin A appeared to have lost the contact and became round. The nuclei had become condensed and were no longer visible.

**Fig. 6:**
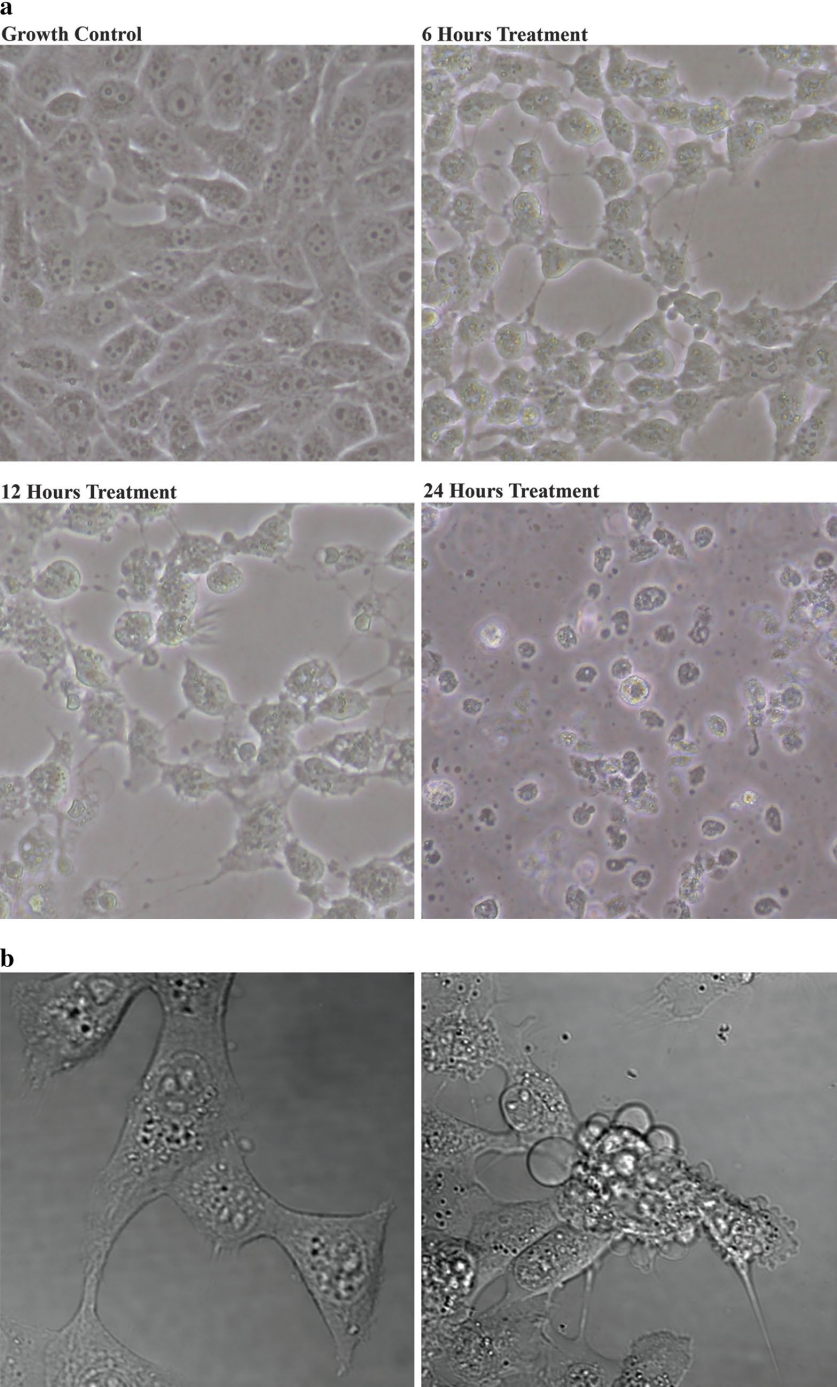
**a** Changes in cell morphology after treatment with pterocellin A at 2000 ng/mL. *Black arrows* pointing at examples of apoptotic features. (Photos taken at ×200 magnification using an inverted Nikon eclipse TS100 microscope with a Nikon COOLPIX 4500 camera). **b** DIC images of a single apoptotic cell from the same experiment. **a** *1* Growth control. *2* Cells treated at 1000 ng/mL for 6 h. (Photos were obtained from an Olympus FV1000 confocal microscope at ×400 magnification)

The presence of apoptotic cells was evident when observed under differential interference contrast (DIC) of a confocal microscope. Membrane blebbing, a characteristic of cells undergoing apoptosis could be seen. Some cells appeared to be ‘shrinking’, and apoptotic ‘spikes’ were also observed. After 24 h treatment, cells were completely detached (Fig. 6a, b).

To investigate the effects of pterocellin A on mitochondria, cells were stained with MitoTracker Red CMXRos and visualised by a laser confocal fluorescent microscope. MitoTracker Red CMXRos is a membrane-permeable fluorescent dye used to label mitochondria. It contains a mildly thiol-reactive chloromethyl moiety that reacts with thiol groups of the proteins in the mitochondria (Poot et al. 1996).

**Fig. 7:**
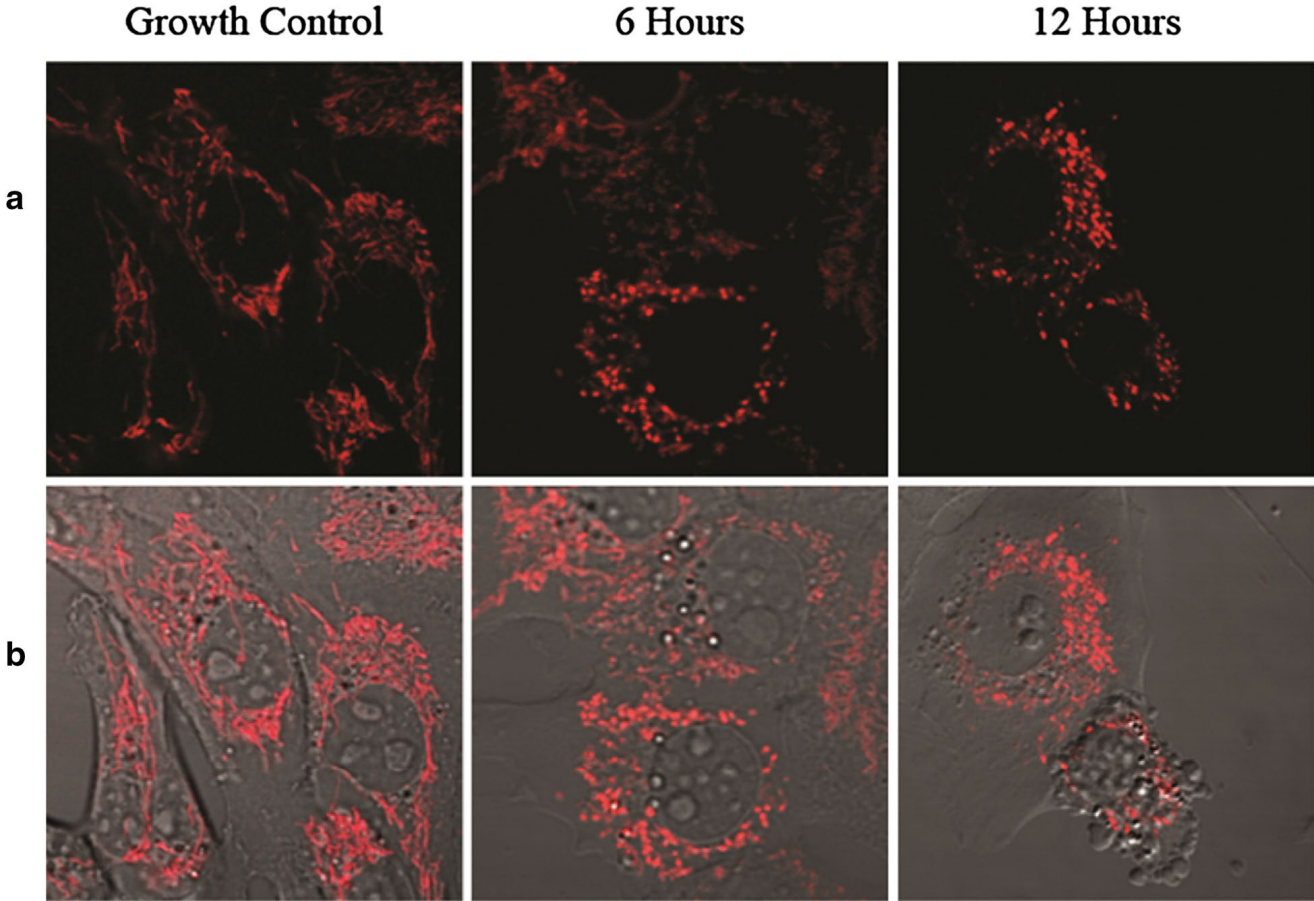
Changes in mitochondrial morphology after pterocellin A treatment at 1000 ng/mL after 6 and 12 h. **a** Mitochondria stained with 50 nM MitoTracker Red CMXRos (Em. 599 nm). **b** Overlay of stained mitochondria and phase contrast images Photos obtained from the Olympus FV1000 laser scanning confocal microscope at ×400 magnification

After treatment with pterocellin A (1000 ng/mL), the mitochondria re-distributed from the cell cytoplasm to the peri-nuclear region after 6 h (Fig. 7). This localisation took place before any notable changes in cell morphology and cell death as shown in the overlay images. Compared to healthy mitochondria that have an elongated tubular formation, the mitochondria of treated cells appeared to be fragmented and ‘swollen’. These changes in the location and morphology of the mitochondria have been reported in the literature including in HeLa cells during UV-induced apoptosis and in pig kidney (PEK) cells after treatment with rotenone, an inhibitor of the respiratory complex I (Modok et al. 2006; Silva 2010; Skulachev et al. 2004; Gao et al. 2001). It was also found that the thread-grain transition occurred after the release of cytochrome *c* from the mitochondria upon induction of apoptosis (Silva 2010). These distinct morphological changes further supported the idea that pterocellin A was an inducer of apoptosis in HeLa cells.

**Fig. 8:**
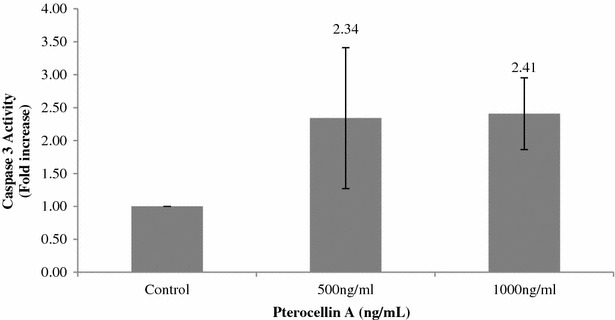
Caspase-3 activity of treated cells compared to control. *Bar graph* showing the mean ± S.E.M, N = 6

Another piece of evidence supporting apoptotic cell death was the activation of caspase-3 detected after treatment by pterocellin A in HeLa cells. Activated caspase-3 is only present upon the induction of apoptosis (Krysko et al. 2008). Upon treatment by pterocellin A (at both 500 and 1000 ng/mL), the level of activated caspase-3 measured by FITC fluorescent staining increased by approximately 2.5-fold compared to untreated control cells (Fig. 8).

## Discussion

A number of MTT and LDH assays were performed during this study. MTT assay was used to determine the cell viability after treatment with pterocellin A. The MTT cell viability assay measures the reduction of MTT, a water-soluble tetrazolium dye, to insoluble formazan crystals. This reduction reaction is dependent on the activity of functioning cellular dehydrogenases in living cells, thus is an indicator of cell viability and mitochondria activity (Berridge et al. 2005). The LDH assay is a colorimetric assay that detects the loss of cell membrane integrity by measuring the release of cytosolic enzyme LDH into extracellular space upon cell lysis. Similar to the MTT assay, the LDH assay involves the enzymatic conversion of a tetrazolium salt, INT [2-(4-iodophenyl)-3-(4-nitrophenyl)-5-phenyl-2H-tetrazolium chloride], into a water-soluble red formazan product. The amount of colour formation is proportional to the amount of cell lysis (Krysko et al. 2008).

Initial experimental data showed that pterocellin A affected cell viability significantly (p < 0.05) within 24 h of treatment in HeLa cells. A longer treatment period did not increase the cytotoxicity, as 100 % of the cells were no longer viable at concentrations above 3125 ng/mL. Interestingly, cells that survived the first 24 h at the lower concentration range had lower relative inhibition values after 48 and 72 h as demonstrated by the IC_50_ values. This suggests that the cells were able to proliferate and overcome the cytotoxic effects after the first 24 h.

The first evidence that suggested that the cells were undergoing apoptosis was demonstrated by the difference in the relative inhibition from the MTT and LDH assays. These assays showed that after 24 h, all cells were killed at 2000 ng/mL according to the MTT data and also by visual observation. However, the LDH data showed only a small increase in the level of detected LDH. The elevated LDH was likely due to a process known as secondary necrosis; this occurs when the apoptotic cells have died but have not been eliminated (Silva 2010).

Time-course experiments of pterocellin A showed that changes in HeLa cells after exposure to pterocellin A were detectable in just after 3 h. Although visually the cell morphology displayed no change, detectable changes were observed in the MTT data. Since MTT is also an indicator of mitochondrial dehydrogenase function (Berridge et al. 2005), this suggests that there was a degree of mitochondria impairment attributed to the cytotoxicity of pterocellin A. Elevated LDH release was only apparent after 12 h treatment at 2000 ng/mL when the cells were almost 90 % inhibited, this further suggest that secondary necrosis took place to some extent after the apoptotic cells had already died.

The trypan blue exclusion experiment indicated that neighbouring cells were indeed taking up apoptotic cells after death as no cells were observed in the cell count, even after collecting supernatant during harvest. However, when observed under the microscope straight after treatment, cell debris could be seen floating in the culture medium after 24 h. These were just cell debris and not intact dead cells, as they did not show up during the trypan blue exclusion cell count. Presence of cell debris indicated that some degree of cell lysis took place in the form of secondary necrosis after apoptotic cells had been phagocytosed.

Further evidence of HeLa cells undergoing apoptosis after exposure to pterocellin A was the morphological changes observed. After 6 h treatment of 2000 ng/mL, it became obvious when observed under the microscope that cells were affected. Treated cells were no longer attached to their neighbouring cells compared to the control, the nucleus was no longer visible and the cells also appeared to ‘shrink’. This shrinking was due to condensation of the nucleus, another character of apoptosis. Additionally, apoptotic spikes and membrane blebbing were also observed within 6 h of treatment. Time-lapse images taken under DIC on a confocal laser microscope displayed movement of the apoptotic blebs as the cells undergo apoptosis (data not shown).

The MitoTracker staining of the mitochondria showed that the mitochondrial morphology changed after 6 h of treatment. In healthy cells, the mitochondria appear elongated and filamentous; after exposure to pterocellin A, distinct changes were observed under a confocal laser microscope. Mitochondria are normally distributed throughout the cytoplasm, however it was found that the mitochondria redistributed from the cell periphery and localised around the nucleus after treatment with pterocellin A. This change took place before changes in cell morphology were observed. The overlay images showed that at 1000 ng/mL after 6 h, the cells still appeared normal compared to the control, however changes in the mitochondria were obvious.

Marine natural products have become important sources in the discovery of antitumour drugs. Cytotoxic natural products that selectively target the apoptotic pathways of cancer cells are particularly of interest in the development of chemotherapeutic agents. There are limited numbers of cytotoxicity studies on marine natural products. Some examples from the literature include: Mycalamide A and pateamine isolated from marine sponges of the *Mycale* genus collected from New Zealand (Hood et al. 2001). In preliminary screening, both compounds have been shown to be apoptotic inducers in H441, LLC-PK1 and SY5Y cells (Hood et al. 2001). Gliotoxin is a natural product produced by the marine fungus *Aspergillus* sp. and has been shown to be cytotoxic to HeLa and human chondrosarcoma (SW1353) cells via apoptotic processes (Nguyen et al. 2014). The induction of apoptosis in HeLa cells by Physcion, an anthraquinone derivative isolated from the marine-derived fungus *Microsporum* sp. has also been reported (Wijesekara et al. 2014). A cyclic peptide microsclerodermin A isolated from the sponge *Amphibleptula* has recently been shown to induce apoptosis in the AsPC-1, BxPC-3 and the PANC-1 cell lines (Guzmán et al. 2015). Heteronemin, a bioactive marine sesterterpene isolated from the sponge *Hyrtios* sp. has been shown to lead to apoptotic cell death in three human cancer cell lines (A549, ACHN, and A498) (Wu et al., 2015).

We have demonstrated for the first time that pterocellin A, a novel alkaloid isolated from the marine bryozoan *Pterocella vesiculosa* collected off the coast of the North Island, New Zealand is cytotoxic to HeLa human cancer cells via apoptotic cellular death processes.

## Conclusions

We present evidence which suggest that pterocellin A could be acting on mitochondria via the intrinsic pathway, however due to the complex nature of the signalling cascades, it is not yet known how the compound enters the cell and which pathway it is interacting with. Out of all the pterocellins, only those with no substitution at position 8, such as pterocellins A and B, exerted the highest antitumour activity against P388 cells (Yao et al. 2003). It could be that the biological interaction of pterocellins is size-dependent. The backbone ring structure could be intercalating with DNA and prompting apoptosis. It is also possible that if pterocellin A is able to target and enter the mitochondria, the mitochondrial DNA (mtDNA) could be more susceptible to damage due to the lack of histone proteins that are normally present in nuclear DNA as regulatory proteins (Brown et al. 1979). Further elucidation of the role of mitochondria as cellular targets for pterocellin A will give insights into the viability of pterocellin A as a lead compound for pharmaceutical development.
